# Temporal and spatial trends in isolated gastroschisis: a population-based study in a middle-income setting

**DOI:** 10.61622/rbgo/2026rbgo53

**Published:** 2026-07-17

**Authors:** Izabelly Oliveira Corsi Nogueira, Ana Sílvia Scavacini Marinonio, Rosa Maria Vieira de Freitas, Monica La Porte Teixeira, Bernadette Cunha Waldvogel, Daniela Testoni Costa-Nobre

**Affiliations:** 1 Universidade Federal de São Paulo Escola Paulista de Medicina São Paulo SP Brazil Escola Paulista de Medicina, Universidade Federal de São Paulo, São Paulo, SP, Brazil; 2 Fundação Sistema Estadual de Análise de Dados São Paulo SP Brazil Fundação Sistema Estadual de Análise de Dados, São Paulo, SP, Brazil

**Keywords:** Gastroschisis, Infant, newborn, Infant mortality, Spatial analysis, Mass screening

## Abstract

**Objective:**

To estimate the prevalence and neonatal mortality of isolated gastroschisis, assess temporal trends, identify risk factors for neonatal death, and describe its spatial distribution in a middle-income setting.

**Methods:**

This population-based study included all live births in São Paulo State, Brazil, from 2004 to 2017. Cases of isolated gastroschisis were identified from live birth and death certificates. Temporal trends in prevalence and neonatal mortality (0-27 days) were analyzed using Prais-Winsten regression to estimate annual percent change (APC). Maternal and neonatal factors associated with neonatal death were examined using Poisson and multivariate logistic regression. Spatial distribution across municipalities was described by quartiles according to maternal residence.

**Results:**

Among 8,441,498 live births, 1664 cases of isolated gastroschisis were identified (prevalence: 1.97 per 10,000). Neonatal mortality rate associated with isolated gastroschisis was 0.35 per 10,000 live births, with a lethality rate of 17.7%. Prevalence (APC 3.0%; 95% CI: –1.3 to 7.5) and mortality (0.6%; –3.5 to 4.8) remained stable over time. Lower gestational age, fewer prenatal visits, and severe growth restriction were independently associated with higher risk of neonatal death. Spatial analysis showed heterogeneous distribution, with most municipalities reporting no cases and higher prevalence and mortality concentrated in few areas.

**Conclusion:**

In this middle-income setting, isolated gastroschisis showed stable but higher prevalence and neonatal mortality than in high-income countries. The heterogeneous spatial pattern suggests regional disparities in risk and outcomes, highlighting the need for targeted public health strategies and further research on gastroschisis epidemiology.

## Introduction

Gastroschisis is a congenital anomaly of the anterior abdominal wall characterized by herniation of abdominal organs through a paraumbilical defect.^(1)^ It is usually an isolated defect, but up to 20% of cases may be associated with other congenital malformations, including both intestinal and extraintestinal anomalies.^(2)^

Worldwide, the prevalence of gastroschisis is estimated at 2.69 per 10,000 live births (1964–2020),^(3)^ and it has increased globally over the last decades.^(4)^ In its isolated form, prevalence has shown wide geographic variation, ranging from 1.2 per 10,000 live births in Poland (1998-2008)^(5)^ to 8.52 per 10,000 live births in Argentina (2009-2013).^(6)^ In the United States, isolated gastroschisis prevalence decreased from 2.86 per 10,000 births in 2014 to 1.55 per 10,000 in 2022.^(7)^

Neonatal mortality rates associated with isolated gastroschisis also vary across regions, from 0.16 per 10,000 live births in United States (2010-2014)^(8)^ to 0.27 per 10,000 live births in Argentina (2009-2013).^(6)^ Lethality is strongly influenced by setting,^(9)^ with rates as low as 8.8% reported in the Netherlands (2002-2010),^(10)^ while substantially higher rates are documented in middle-income countries.^(6,9)^

In Brazil, a middle-income country, a recent population-based study reported an overall prevalence of isolated gastroschisis of 2.15 per 10,000 live births, with a 23% increase between 2007 and 2020 and significant regional variation.^(11)^ Data on neonatal mortality are scarce, but in the state of Rio de Janeiro, Brazil, the mortality rate of infants under one year of age with isolated gastroschisis was 0.74 per 10,000 live births (2004–2014).^(12)^ Hospital-based studies also show variable lethality, ranging from 12.7% in São Paulo^(10)^ to 33.8% in Espírito Santo, another Southeastern Brazilian state (2000–2018).^(13)^

São Paulo State, located in southeastern Brasil, is the most populous and economically developed state in the country, comprising 645 municipalities and approximately 44 million inhabitants.^(14)^ It has a high-quality vital statistics system, capturing approximately 99.5% of civil registry data.^(15)^ Despite these characteristics, there are no population-based studies specifically addressing the epidemiology of isolated gastroschisis in São Paulo. Such information is essential to guide public health policies and improve neonatal outcomes.

Therefore, we conducted a population-based study including all live births in the State of São Paulo between 2004 to 2017 aiming at: 1) Estimate the prevalence and neonatal mortality associated with isolated gastroschisis and analyze their temporal trends; 2) Identify maternal and neonatal factors associated with neonatal death of live births with isolated gastroschisis; and 3) Describe the spatial distribution of isolated gastroschisis prevalence and mortality across municipalities.

## Methods

This was a population-based study including all live births from mothers residing in the State of São Paulo, Brazil, between January 1, 2004, and December 31, 2017. Eligible newborns had a gestational age ≥22 weeks and a birth weight ≥400 grams. Newborns with unknown gestational age and birth weight were excluded.

The database was constructed by deterministic linkage of live birth and death certificates by the State Data Analysis System Foundation (Fundação SEADE), the official institution responsible for managing these records in the State of São Paulo. Live births with gastroschisis (ICD-10/WHO: Q79.3) were identified from either the live birth certificate or by the presence of the code in any line of the death certificate. Isolated gastroschisis was defined as the presence of gastroschisis without any other congenital anomaly (ICD-10/WHO codes Q00 to Q99, except Q79.3).

Temporal trends in the prevalence and neonatal mortality (0-27 days) of isolated gastroschisis were analyzed using the Prais-Winsten regression model, which estimates the annual percent change (APC) with 95% confidence intervals (CI), and classifies trends as stationary, increasing, or decreasing. Prevalence rates of isolated gastroschisis per 10,000 live births and neonatal mortality rates associated with isolated gastroschisis per 10,000 live births were calculated for each of the 645 municipalities, according to place of birth.

Newborns with isolated gastroschisis who died within 27 days after birth were compared to survivors (alive at 27 days). Maternal and neonatal variables were obtained from live birth certificates and/or death certificate. The following demographic characteristics were compared between both groups: maternal age (<20, 20-34, and ≥35 years), marital status (single mother yes/no), maternal schooling (≤7, 8-11, ≥12 years), parity (primiparous /multiparous), number of prenatal care visits (0-6 /≥7), type of pregnancy (singleton/multiple), delivery mode (vaginal/cesarean), gestational age (22-27, 28-31, 32-36, and ≥37 weeks), birth weight (<1500g, 1500-2499g, and ≥2500g), sex (male/female), severe birth restriction (yes/ no), 1^st^ minute Apgar score (<7/≥7) and 5^th^ minute Apgar score (<7/≥7). Because gestational age was only available in categories, severe birth restriction was defined as birth weight <10^th^ percentile of the lowest gestational age within each category, according to the Intergrowth-21^st^.^(16)^ No inferential statistical tests were performed, as the analysis included the entire population rather than a sample.

To investigate factors associated with neonatal death among infants with isolated gastroschisis, maternal and neonatal characteristics were initially analyzed using Poisson regression adjusted only for year of birth to estimate incidence rate ratios (IRRs). Variables significant at *p*<0.05 in the univariate analysis were then included in a multivariate logistic regression model. Birth weight was excluded from the multivariate model due to collinearity with gestational age, and Apgar scores were excluded because of missing data from 2014 to 2017.

Prevalence and neonatal mortality rates of isolated gastroschisis from 2004 to 2017 were mapped by quartiles across São Paulo municipalities, considering maternal municipality of residence. Both outcomes were consistently assigned based on maternal residence, regardless of place of birth or death, to minimize potential bias related to referral patterns. Statistical analyses were performed using Stata 18® (StataCorp LLC, College Station, TX, USA), and spatial distribution maps were generated with TerraView® version 4.2.2 (Instituto Nacional de Pesquisas Espaciais, São José dos Campos, Brazil). The study was approved by the Research Ethics Committee of the Federal University of São Paulo under approval number 6.614.617 and CAAE # 75345223.9.0000.5505.

## Results

**Figure 1:**
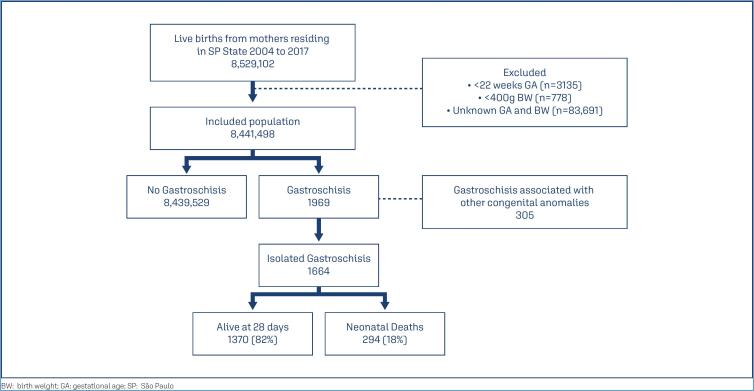
Study flow diagram

From 2014 to 2017, there were 8,529,102 live births from mothers residing in the State of São Paulo (Figure 1). Among these, 8,441,498 were included in the study and 1969 livebirths were diagnosed with gastroschisis according to ICD-10. Among them, 1664 (84.5%) presented isolated gastroschisis and 294 (18%) of them died in the neonatal period.

**Figure 2:**
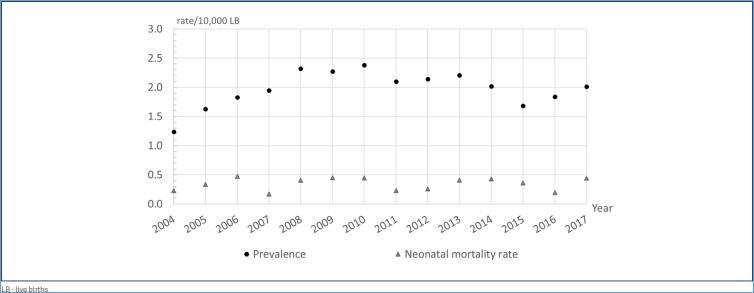
Trend of prevalence and neonatal mortality rate of isolated gastroschisis (per 10,000 live births)

The prevalence of isolated gastroschisis was 1.97 per 10,000 live births, and the neonatal mortality rate was 0.35 per 10,000 live births during the study period. The lethality rate of isolated gastroschisis in our study was 17.7% (294/1964) in the first 27 days of life. Over the years, the prevalence and the neonatal mortality rate of infants with isolated gastroschisis varied (Figure 2), and the temporal trend for both was not significant: prevalence APC 3.0% (95%CI: -1.3 to 7.5) and neonatal mortality rate APC 0.6% (95%CI: -3,5 to 4.8).

Compared with survivors, neonates with isolated gastroschisis who died during the neonatal period were born to younger mothers who had fewer prenatal visits; they also had lower gestational age and birth weight, a higher incidence of severe growth restriction, and lower Apgar scores at both one and five minutes (Table 1).

**Table 1:** Comparison of the demographic characteristics between live births with isolated gastroschisis alive at 28 days and those who died during the neonatal period

Variables		Alive at 28 days n(%)	Gastroschisis-associated neonatal deaths n(%)
Maternal age (years)			
	<20	611(44)	153(52)
	20–34	735(54)	136(46)
	≥35	23(2)	5(2)
Maternal schooling (years)			
	≤7	253(26)	67(33)
	8–11	639(66)	119(58)
	≥12	75(8)	18(9)
Single mother			
	Yes	704(73)	149(73)
Prenatal care visits			
	<7	521(39)	140(49)
Pregnancy			
	Multiple	17(1)	3(1)
Delivery			
	Vaginal	270(20)	69(23)
Gestational age (weeks)			
	22–27	8(1)	10(3)
	28–31	42(3)	18(6)
	32–36	646(48)	145(50)
	≥37	646(48)	119(41)
Birth weight (grams)			
	<1500	45(3)	35(12)
	1500–2499	790(58)	185(63)
	≥2500	534(39)	74(25)
Sex			
	Male	715(52)	154(52)
Severe birth restriction			
	Yes	215(16)	70(24)
	1^st^ minute Apgar score		
	<7	268(28)	85(43)
5^th^ minute Apgar score			
	<7	41(4)	24(12)

n - number

In the univariate Poisson regression analysis adjusted only for year of birth, maternal age under 20 years, attending up to six prenatal visits, lower gestational age, lower birth weight, severe birth weight restriction, and Apgar scores below 7 at the first and fifth minutes were associated with a higher incidence of neonatal death in infants with isolated gastroschisis (Table 2). In the final multivariate model, compared with infants with gestational age of 37 weeks or more, those with gestational age of 22-27 weeks had a higher risk of death (IRR 3.80; 95% CI 2.19, 6.57), followed by those with 28-31 weeks (IRR 2.36; 95% CI 1.54, 3.62) and 32-36 weeks (IRR 1.51; 95% CI 1.14, 2.00). Attending fewer prenatal visits (0-6 vs. ≥ 7) (IRR 1.26; 95% CI 1.02, 1.55) and having severe birth weight restriction (IRR 2.03; 95% CI 1.50, 2.73) were also independently associated with higher risk of neonatal death for live births with isolated gastroschisis.

**Table 2:** Univariate Poisson regression models (adjusted only for year of birth) for neonatal death in infants with isolated gastroschisis

Variables		IRR	95%CI
Maternal age (Years)			
	<20	1.28	1.04 to 1.58
	20-34	Reference	
	≥ 35	1.14	0.51 to 2.60
Maternal schooling (year)			
	≤7	1.08	0.68 to 1.72
	8 to 11	0.81	0.52 to 1.27
	≥ 12	Reference	
Single mother		1.04	0.78 to 1.38
Prenatal care visits <7		1.42	1.15 to 1.75
Multiple gestation		0.85	0.30 to 2.42
Vaginal delivery		1.20	0.94 to 1.52
Gestational age (weeks)			
	22-27	3.57	2.29 to 5.57
	28-31	1.93	1.27 to 2.94
	32-36	1.18	0.94 to 1.47
	≥ 37	Reference	
Birth weight (grams)			
	<1500	3.59	2.59 to 4.99
	1500 to 2499	1.56	1.21 to 2.00
		Reference	
Male sex		1.01	0.82 to 1.24
Severe growth restriction		1.49	1.18 to 1.89
1^st^ minute Apgar score <7		1.72	1.34 to 2.22
5^th^ minute Apgar score <7		2.33	1.65 to 3.29

IRR - incidence rate ratio; CI - Confidence interval

**Figure 3:**
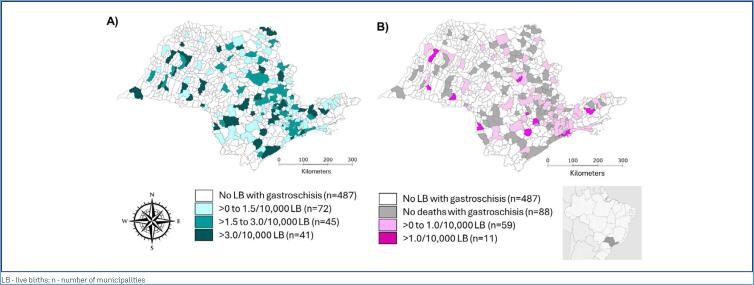
Spatial distribution of A: prevalence (per 10,000 live births) and B: neonatal mortality rate (per 10,000 live births) of isolated gastroschisis

A total of 487/645 (77%) municipalities did not report any live births with isolated gastroschisis. Among the remaining 158 municipalities, the total number of live births with isolated gastroschisis ranged 1 to 610, with prevalence rates varying between 0.25 to 152/10,000 live births (Figure 3A). Among the 645 São Paulo State municipalities, 575/635 (89%) did not have infants with isolated gastroschisis that died between 0-27 days (487 municipalities without live births with isolated gastroschisis and 88 municipalities with live births with isolated gastroschisis but no neonatal death of infants with the anomaly) during the study period. Among the remaining 70 municipalities, the mortality rate of neonates with isolated gastroschisis ranged from 0.1 to 5.5/10,000 live births (Figure 3B).

## Discussion

This populational-based study conducted in São Paulo state, Brazil, between 2004 and 2017 showed a prevalence of isolated gastroschisis of 1.97 per 10,000 live births, and a neonatal mortality rate of 0.35 per 10,000 live births, corresponding to a lethality of 17.7%. Both prevalence and mortality rates had a stable trend during the study period. Lower gestational age, fewer prenatal care visits, and severe birth restriction were independently associated with neonatal death among infants with isolated gastroschisis. Spatial analysis revealed a heterogenous distribution across municipalities, with a concentration of higher prevalence and mortality rates in few areas, whereas most municipalities reported no cases.

The prevalence identified in our study was slightly lower than that reported by Calderon et al. (2.15 per 10,000 live births) for São Paulo State between 2005 and 2016,^(17)^ and higher than the 1 per 10,000 live births observed in Paraíba in 2021.^(18)^ However, neither of these previous studies restricted their analysis to isolated gastroschisis. The differences observed may reflect the higher concentration of medical services, improved diagnosis of congenital anomalies,^(19)^ and better quality of health records in São Paulo State.^(20)^ Since 2005, the municipality of São Paulo has implemented continued education and monitoring initiatives aiming to improve the diagnosis and the report of congenital anomalies on live birth certificates. These sustained efforts have contributed to enhanced data quality, reducing missing information and increasing the notification accuracy of neonates with congenital anomaly.^(19)^

Unlike the previously described increase in the temporal trend of gastroschisis by 2.6% per year in São Paulo State between 2005 and 2016,^(17)^ our analysis demonstrated a stable pattern from 2004 to 2017 with minor annual fluctuations. This stability may be related to the fact that the present study included only isolated cases of gastroschisis, a visibly identifiable anomaly at birth with a well-established diagnosis, which reduces the influence of variations in data quality.^(21)^ Additionally, temporal improvements in data quality, completeness, and reporting practices may have occurred over the study period. Such changes can enhance case detection and registration, and should be considered when interpreting temporal trends, as part of the observed patterns may reflect ascertainment bias rather than a true change in incidence. It is also important to consider that, in settings where surveillance relies on live birth data, observed prevalence may be influenced not only by true incidence but also by changes in fetal survival. Improvements in prenatal diagnosis and perinatal care may reduce fetal mortality and increase the likelihood of live birth among affected pregnancies, potentially masking underlying epidemiological trends.

Despite advances in neonatal care and implementation of public health initiatives for congenital anomaly prevention in Brazil,^(18)^ neonatal mortality and lethality rates for isolated gastroschisis in this study (0.35 per 10,000 live births and 17.7%, respectively) remain considerably higher than those reported in high-income countries, where neonatal lethality rarely exceeds 10%.^(10)^ Multiple systemic factors may contribute to those disparities, including suboptimal prenatal care quality, lack of integration between healthcare levels, absence of structured referral pathways, and inconsistent application of management protocols.^(22-24)^ Additionally, inequities in the distribution of neonatal intensive care units and specialized surgical services across municipalities likely exacerbate regional differences in outcomes.^(12,25)^

Adequate prenatal care and the early antenatal diagnosis of gastroschisis are key to improving outcomes. Ultrasonographic detection enables referral to tertiary centers equipped for neonatal surgery and advanced intensive care.^(24)^ Delivery in such centers allows prompt surgical intervention, shorter hospitalization, lower infection rates, and higher survival.^(26,27)^ In Shanghai (2000-2012), being born outside a referral center increased lethality by 33%.^(27)^ Similarly, the implementation of a multidisciplinary management protocol in a São Paulo referral center reduced the lethality from 34% (1989-2002) to 25% (2003-2013).^(28)^ Protocol-based care standardizes management and ensures coordinated action among obstetric, neonatal, and surgical teams.^(26)^ In contrast, the absence of unified clinical pathways results in variability in treatment approaches,^(8)^ likely contributing to the persistently high mortality observed.

The spatial distribution showed that mothers of newborns with gastroschisis were living in approximately 25% of the state's municipalities, indicating a wide geographical spread of cases. Moreover, prevalence varied considerably across municipalities. Lower prevalence rates observed in some areas may be related to underreporting,^(29)^ whereas higher prevalence rates could result from the small number of live births in certain municipalities during the study period. Additionally, the influence of socioeconomic inequality should be considered for the prevalence and lethality variation. In 2010, São Paulo's Municipal Human Development Index (IDHM) ranged from 0.639 to 0.862, demonstrating persistent socioeconomic heterogeneity across the state.^(30)^ Lower socioeconomic conditions may be associated with behavioral and environmental factors, nutritional deficiencies, and limited access to healthcare, all of which are determinants that may influence gastroschisis prevalence.^(31)^ Furthermore, gastroschisis outcomes are strongly influenced by healthcare access, and by the availability and quality of neonatal surgery and postoperative support. Lethality with gastroschisis is higher in lower socioeconomic settings, where healthcare resources are often constrained and timely access to specialized care may be limited.^(9,26)^

This study has several limitations. Due to the unavailability of data for some key variables from live birth and death certificates after 2017, resulting from operational limitation in data processing, more recent data could not be included in the analysis, as this could compromise consistency and reliability. Therefore, data loss and inconsistencies in subsequent years limited the study period. The data originate from live birth and death certificates, which are subject to information bias, as they rely on accurate diagnosis reporting. Additionally, the classification of cases as isolated gastroschisis was based on available registry information, which may have incomplete reporting of associated anomalies. Therefore, some cases classified as isolated may have had additional anomalies that were not recorded. Gestational age in this study was obtained from administrative records and categorized into intervals rather than recorded as exact weeks, which may reduce its accuracy compared with birth weight. As a result, it was not possible to apply the standard definition of small for gestational age based on week-specific birth weight percentiles. To address this limitation, severe growth restriction was defined using the 10th percentile of the lowest gestational age within each category, according to Intergrowth-21st standards. However, this approach may have led to misclassification and should be interpreted with caution.

Moreover, the absence of detailed clinical data – such as surgical management, timing of intervention, and postoperative complications – precludes a more granular understanding of determinants of mortality. The analysis also excludes stillbirths, limiting comparison with studies including all pregnancy outcomes. In Brazil, pregnancy termination for gastroschisis is not legally permitted and is therefore unlikely to influence prevalence estimates. However, this should be considered when comparing our findings with studies from settings where termination of pregnancy for congenital anomalies is allowed, as this may reduce the number of affected live births and impact prevalence estimates. Despite these limitations, this study represents a population-based analysis of temporal trends in isolated gastroschisis, prevalence and neonatal mortality in a state of a middle-income country encompassing over 8 million live births. These findings may support more specific studies on the epidemiology of gastroschisis in São Paulo State and may contribute to guiding strategic planning and improving the organization of neonatal care across the region.

## Conclusion

In conclusion, this population-based study identified stable but high prevalence and neonatal mortality rates of isolated gastroschisis in São Paulo State between 2004 and 2017. Low gestational age, limited prenatal care and severe birth restriction were associated with neonatal deaths in this population. The wide geographic dispersion of maternal residence underscores persistent inequalities in healthcare access and infrastructure. These findings highlight the need for strengthening prenatal diagnosis, referral networks, and standardized management protocols to improve outcomes and reduce mortality associated with gastroschisis in a middle-income setting.

## Data Availability

The research data are described in the article presented.
